# Mid-term results of a new femoral prosthesis using Ti-Nb-Sn alloy with low Young’s modulus

**DOI:** 10.1186/s12891-021-04879-1

**Published:** 2021-11-26

**Authors:** Daisuke Chiba, Norikazu Yamada, Yu Mori, Masamizu Oyama, Susumu Ohtsu, Yoshiyuki Kuwahara, Kazuyoshi Baba, Hidetatsu Tanaka, Toshimi Aizawa, Shuji Hanada, Eiji Itoi

**Affiliations:** 1 Department of Orthopaedic Surgery, Tohoku University Graduate School of Medicine, 1-1 Seiryo-machi, Aoba-ku, Sendai, Miyagi 980-8574 Japan; 2 Department of Orthopaedic Surgery, Sendai Red Cross Hospital, 2-43-3 Yagiyamahoncho, Taihaku-ku, Sendai, Miyagi 982-8501 Japan; 3 Department of Orthopaedic Surgery, Osaki Citizen Hospital, 3-8-1 Furukawahonami, Osaki, Miyagi 989-6183 Japan; 4 Institute for Materials Research, Tohoku University, 2-1-1 Katahira, Aoba-ku, Miyagi 980-8577 Japan

**Keywords:** Total hip Arthroplasty, Low Young’s modulus, Stress shielding, Ti-Nb-Sn alloy; β type titanium alloy

## Abstract

**Background:**

This study was performed to investigate the mid-term results of Ti-Nb-Sn (TNS) alloy stem with a low Young’s modulus.

**Methods:**

This study was a multicenter prospective cohort study. A total of 40 primary total hip arthroplasties performed between April 2016 and September 2017 was enrolled in this study. With the unique functional gradient properties by heating treatment, the strength of the proximal portion was enhanced, while the distal portion maintained a low Young’s modulus. The surgeries were performed through the posterolateral approach using the TNS alloy stems. Radiographs were taken from immediately after surgeries until 3 years, and stress shielding and subsidence of the stems were evaluated. The incidences of the stem breakage were also assessed. Clinical assessments were performed using Japanese Orthopaedic Association (JOA) and Japanese Orthopaedic Association Hip Disease Evaluation Questionnaire (JHEQ) scores.

**Results:**

Among the 40 enrolled patients, 36 patients were female and 4 were male. At 3 years after surgery, there were no radiologic signs of loosening, subsidence, or breakage of the stem. Stress shielding was observed in 26 hips (65%). Of 26 hips, 16 hips (40%) were grade 1 and 10 hips (25%) were grade 2. There was no advanced stress shielding. The JOA and JHEQ scores significantly improved compared with the preoperative scores.

**Conclusion:**

The current study using a new TNS alloy femoral stem showed good clinical outcomes at 3-year follow-up. Radiologically, there was no loosening or subsidence of the stem. The mild stress shielding was observed in 65% of patients.

**Trial registration:**

Current Controlled Trials ISRCTN21241251.

The date of registration was October 26, 2021.

Retrospectively registered.

## Background

The good long-term results of total hip arthroplasty (THA) have been reported in patients with hip osteoarthritis. Previous long term follow-up studies demonstrated that the improvements of hip pain, functions and quality of life were achieved by THA [1, 2]. Furthermore, the long-term results of cementless femoral stem have demonstrated favorable results in clinical and radiological assessments, same as cemented femoral stem. Therefore, THA with cementless stem has prevailed worldwide [3–7].

However, there are still several issues to be solved in THA with cementless stem. One of the major problems is the stress shielding due to inadequate load-stress distribution. Stress shielding refers to the bone atrophy and loss of bone density as results of load stress being removed from the bone by the femoral stem [8, 9]. The bone atrophy and loss of bone mineral density by the stress shielding increase the risk of periprosthetic fracture [10, 11]. As the number of THA performed in elderly patients with osteoporosis has been increasing, the prevention of periprosthetic fracture is a very important issue. The etiology of stress shielding is considered multifactorial such as surface texture, geometry, and stiffness of the stem [12]. Currently, femoral stems are made with Ti-6Al-4V alloy with a Young’s modulus of 110 GPa and a tensile strength to 860 MPa. Ti-6Al-4V alloy has a good biocompatibility and resistance to corrosion. In contrast, the Young’s modulus of the human cortical bone ranges between 10 GPa and 30 GPa [13]. This mismatch of elasticities between the femoral stem and the cortical bone is considered one of the major causes of stress shielding.

To solve the mismatch of elasticity between the stem and the bone, the Robert Mathys (RM) isoelastic stem was developed as a low elastic stem to avoid stress shielding [14–16]. However, the results of isoelastic RM stem were demonstrated to be unacceptably poor. The deficiency of primary fixation was believed to be the main reason for the high failure rate [17]. In contrast, Hanada et al. developed a new β type titanium alloy, Ti-33.6% Nb-4% Sn (TNS) alloy with the Young’s modulus as low as 40 GPa. Not only the low Young’s modulus, but also the modifiability of material properties by heat are the great assets of the TNS alloy. The researchers developed a new cementless stem of TNS alloy with the functional gradient properties of Young’s modulus and strength by heating treatment [18]. Furthermore, the safety and biocompatibility were demonstrated in the previous studies [18, 19]. TNS alloy stem with high strength in the proximal part and low Young’s modulus in the distal part are considered ideal for the prevention of fatigue failure and stress shielding.

The authors hypothesized that a TNS alloy stem with functional gradient properties of Young’s modulus and strength could prevent the stress-shielding problem and improve the postoperative outcome of cementless THA. The purpose of this study was to investigate the mid-term results of TNS alloy stem.

## Methods

### Patients

The study was a multicenter, open-label, single-arm clinical trial. The approval of Clinical Research Ethics Committee of Tohoku University Hospital was obtained before the initiation of this study (Approval number: #201506–1). From April 2016 to September 2017, a total of 425 total hip arthroplasties were performed at our institutions, and 40 primary total hip arthroplasties using the TNS alloy stem were performed. These 40 patients were enrolled in this study. The inclusion criteria for this study were patients over 20 years of age who had a preoperative diagnosis of osteoarthritis, avascular necrosis, or rheumatoid arthritis, and consent to participate in the study. Patients who did not wish to participate in the clinical study did not undergo total hip arthroplasties with TNS alloy stems. The exclusion criteria were previous operation (total hip arthroplasty, osteotomy, tenotomy around hip joint), bilateral hip disorder, rheumatoid arthritis of Charnley category C (multiple joint disease or other disease limiting mobility) [20], past history of deep venous thrombosis or pulmonary embolism, metal allergy, severe obesity (Body Mass Index > 35.0 kg/ m^2^), severe diabetes mellitus, and infection around hip joint. The surgical cases with TNS femoral stems were selected based on the choices and consent of patients who met the inclusion criteria. In our institutes, total hip arthroplasties with antero-lateral supine hip approach using short-designed stems were mainly performed. There were few cases operated with posterolateral hip approach using conventional metaphyseal filling type stems of Ti-6Al-4V alloy in the study period. Therefore, the control cases were not able to be established in this study.

### Characteristics of a new TNS alloy stem

**Fig. 1:**
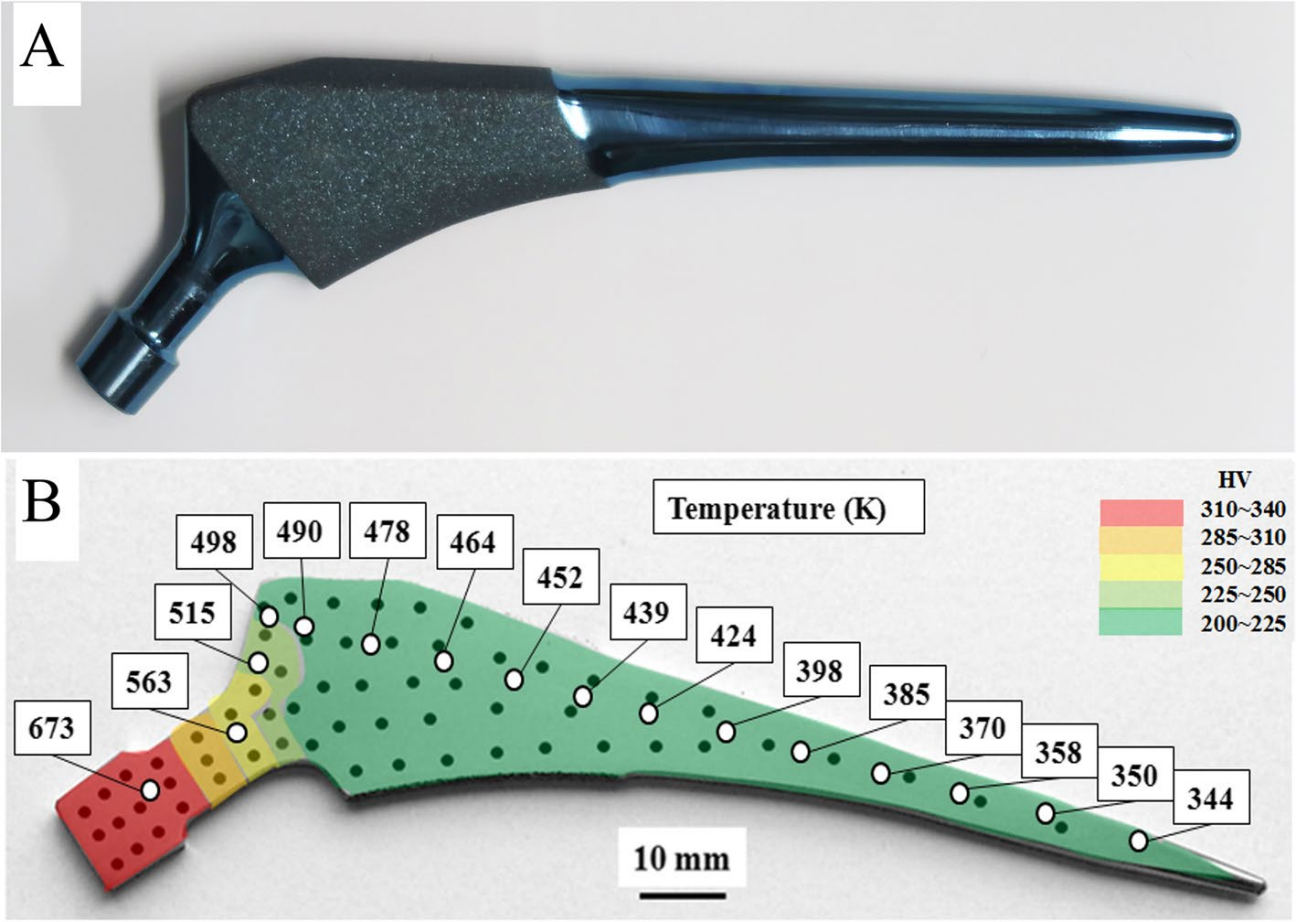
**A** Photograph of TNS alloy stem. **B** Schematic model of the low thermal conductivity and Vickers hardness of TNS alloys. The white circles show the actual measured values at each site, where the temperature (K) of the TNS alloy stem due to heat treatment. Color mapping of the stem is shown for Vickers hardness (HV) distribution. The black circles show the measured Vickers hardness at each site. HV: Hardness of Vickers

TNS alloy is a new material with a low Young’s modulus of 40 GPa [18]. TNS alloy has characteristics that the Young’s modulus and strength can be modified by heating treatment and a low thermal conductivity. The fabrication of the TNS alloy stem and the changes of Young’s modulus and tensile strength of the TNS alloy stem with heat treatment have been described in previous studies in detail, and the TNS alloy stem used in this study was fabricated according to the method reported in a previous study [18, 21, 22]. As briefly described, when stem neck was heated locally, it made the temperature gradation, high temperature in the proximal and low temperature in the distal portions of the stem. Thereby local heat treatment at stem neck at 673 K for 5 h increased its Young’s modulus and strength in proximal part resulted in a gradual decrease in the Young’s modulus from the proximal to the distal end. The authors made a high-performance stem from the TNS alloy with high strength in the proximal part and low Young’s modulus in the distal part of the stem. Figure 1A shows the appearance of the TNS alloy stem. The TNS alloy stem is cementless tapered proximal fixation stem. According to the classification of Khanuja [23], it is classified as double wedge metaphyseal filling stem. Over the proximal one third of the stem, there is a circumferential rough surface by sandblasting. The surface of distal two third is polished. Vickers hardness has been reported to be correlated with Young’s modulus [24]. Figure 1B shows the temperature gradient and Vickers hardness measurements after 5 h of heat treatment at stem neck at 673 K. The white circles show the actual measured values at each site, where the temperature change of the TNS alloy stem due to heat treatment was measured from proximal to distal. Color mapping of the stem is shown for Vickers hardness distribution. The black circles show the measured Vickers hardness values reflecting the change in Young’s modulus after heat treatment at each site. The neck part of the TNS alloy stem had sufficient strength of more than 250 hardness of Vickers (HV). In contrast, the temperature was 344 K in the distal part of TNS alloy stem due to the gradient function property. There was no increase in Vickers hardness in the distal portion of the TNS alloy stem than the neck where the temperature was kept below 500 K.The TNS alloy stem was manufactured and provided by Mizuho Co. Tokyo, Japan. The cost of the clinical trials was covered by Mizuho Co. Tokyo, Japan.

### Surgery and rehabilitation

All surgeries were performed at three institutions by 6 orthopaedic surgeons through the posterolateral hip approach. The femur was scraped with a hand-powered reamer and a broach. The TNS alloy stem was inserted with the press-fit technique. As acetabular component, ARC HA cup (Mizuho Co., Tokyo, Japan) was used in all patients. All the patients were allowed to begin full weight bearing with use of an assistive device on the first postoperative day.

### Radiographical evaluation

Anteroposterior radiographs of bilateral hips and lateral radiographs of affected hip with calibration measure were taken before the surgery, immediately after the surgery, at 3 weeks, 6 weeks, 3 months, 6 months, 1 year, and 3 years postoperatively. The incidences of stress shielding were assessed with radiographs at 3 years using the classification of Engh [25]. Stem subsidence was defined as the inferior migration of the stem by more than 2 mm from the initial postoperative radiograph. Stem subsidence was evaluated at every time point. Also, the stem breakage was assessed on radiographs at every follow-up. Radiological assessment of stress shielding, subsidence, and breakage of the stem was performed by two independent orthopaedic surgeons.

### Clinical assessment

The Japanese Orthopaedic Association (JOA) hip scores were used to assess the clinical outcomes before the surgery, 6 weeks, 3 months, 6 months, and 1 year postoperatively. The JOA hip score is a 100-point scale that comprises the subcategories of pain (Pain: 40 points), range of motion (ROM: 20 points), ability to walk (Gait: 20 points) and activities of daily living (ADL: 20 points) [26]. To assess the patient-reported outcome measures, the Japanese Orthopaedic Association Hip Disease Evaluation Questionnaire (JHEQ) was used before the surgery, 3 months, 6 months, and 1 year postoperatively. The JHEQ consists of pain (28 points), movement (28 points) and mental (28 points) subscales, with 84 points being the best outcome [27].

### Statistical analysis

All results were expressed as the mean ± standard deviation. For the evaluation of the differences between the preoperative and postoperative JOA hip scores and JHEQ scores, Bonferroni correction and Mann-Whitney U test were applied to evaluate statistical differences using a data analysis software (JMP 15, SAS Institute Japan, Tokyo, Japan). If number of the null hypothesis was k in the multiple comparisons, level of significance α was adjusted at α / k for each comparison by Bonferroni correction. A value of *p* < 0.05 / k was considered statistically significant. Kappa coefficients for reproducibility of scores between examiners for grading of Engh’s classification were calculated using SPSS version 21 (IBM, Armonk, NY, USA) to assess the reliability of the measurements.

## Results

### Patients characteristics

Among the 40 patients enrolled, 36 patients were female and 4 were male. The average age at operation was 64.2 ± 10.7 years. The mean follow-up period for these patients after the surgery was 3.7 ± 0.8 years. The mean weights of female and male patients at surgery were 56.5 ± 10.2 kg and 73.4 ± 5.6 kg, respectively. The mean heights of female and male patients were 151.6 ± 7.2 cm and 172.5 ± 8.1 cm, respectively. The mean body mass index was 24.6 ± 4.1 kg/m^2^. In terms of diagnosis, 35 patients were osteoarthritis and the rest 5 patients were avascular necrosis. Patients with rheumatoid arthritis were not included (Table 1).

**Table 1:** Demographics of the Patients

Variable	
Number of patients (hips)	40
Gender Female: Male, n (%)	36 (90%): 4 (10%)
Mean age at surgery (years)	64.2 ± 10.7
Mean follow-up period (years)	3.7 ± 0.8
Pre-operative diagnosis, n (%)	
Osteoarthritis	35 (87.5)
Avascular necrosis	5 (12.5)
Body Mass Index (Kg/m2)	24.6 ± 4.1

Results are the mean with a standard deviation in parenthesis

### Radiological evaluation

**Fig. 2:**
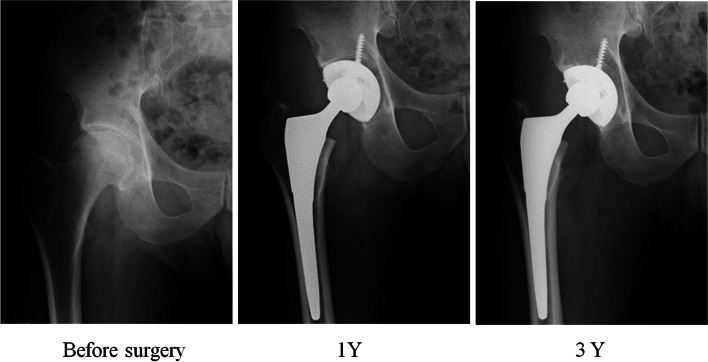
Radiographs of the representative case without stress shielding. **A** Before surgery. **B** 1 year after surgery. **C** 3 year after surgery

At every time point, there were no radiographical signs of loosening, subsidence and breakage of the stem. At 3 years after surgery, stress shielding was observed in 26 hips (65%). Of these 26 hips, 16 hips (40%) were grade 1 and 10 hips (25%) were grade 2 according to Engh’s classification. Severe stress shielding in grade 3 or 4 was not observed (Table 2). Kappa coefficient for reproducibility of scores between examiners for grading of Engh’s classification was 0.75. The radiographs of the representative case without stress shielding were demonstrated in Fig. 2.

**Table 2:** Stress shielding according to the Engh’s classification at 3-year follow-up

Engh’s classification	Number of patients (Hips)	Ratio (%)
None	14	35
Grade 1	10	25
Grade 2	16	40
Grade 3	0	0
Grade 4	0	0

### Clinical evaluation

**Fig. 3:**
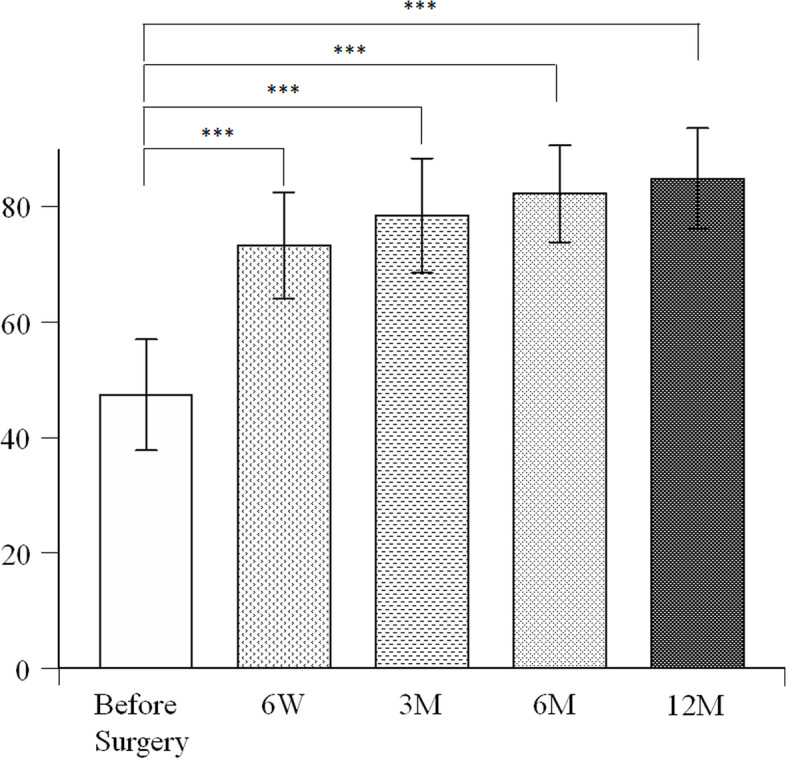
Comparison of Japanese Orthopaedic Association Hip Scores before surgery, and 6 weeks, 3 months, 6 months, and 1 year after surgery. ***P < 0.00025 by Mann-Whitney U test with post-hoc by Bonferroni correction for 4 comparisons

**Fig. 4:**
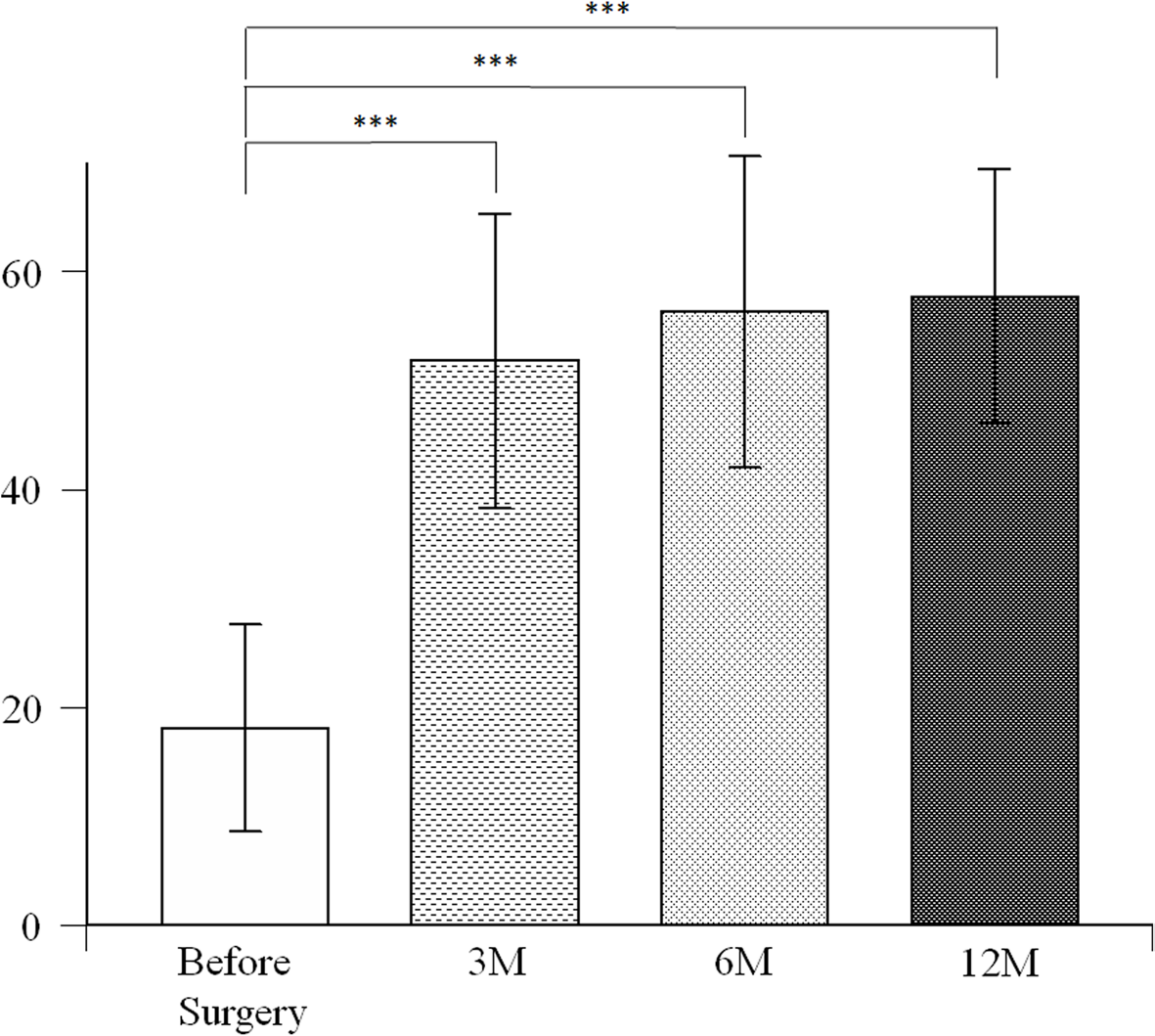
Comparison of Japanese Orthopaedic Association Hip Disease Evaluation Questionnaire before surgery, and 3 months, 6 months, and 1 year after surgery. ***P < 0.00055 by Mann-Whitney U test with post-hoc by Bonferroni correction for 3 comparisons

The JOA scores were 47.4 ± 9.6 points before surgery, which improved to 73.3 ± 9.2 points at 6 weeks, 78.5 ± 9.9 points at 3 months, 82.3 ± 8.4 points at 6 months and 84.9 ± 8.6 points at 1 year. Compared with the preoperative JOA scores, the postoperative scores improved significantly at 6 weeks, 3 months, 6 months, and 1 year (*p* < 0.0001, *p* < 0.0001, *p* < 0.0001, *p* < 0.0001, respectively) (Fig. 3). The preoperative JHEQ scores were 18.1 ± 9.5 points, which improved to 51.8 ± 13.5 points at 3 months, 56.9 ± 14.3 points at 6 months, 57.5 ± 11.7 points at 1 year after surgery. Compared with the preoperative JHEQ scores, the postoperative scores improved significantly at 3 months, 6 months, and 1 year (*p* < 0.0001, *p* < 0.0001, *p* < 0.0001, respectively) (Fig. 4). No adverse event including allergic reaction was observed. There were no periprosthetic infection, dislocation, or revision surgery.

## Discussion

The current study showed good clinical outcomes at 3 years using this new TNS alloy femoral stem. Radiological results also showed good fixation in all cases without loosening and subsidence of the stem. There were no complications such as the breakage and corrosion of the stem, and no safety issues were encountered.

The incidence of stress shielding at 3 years was 65% in this study. The grade of stress shielding was mild, up to grade 2, and there was no occurrence of advanced stress shielding. In contrast, previous studies using the same design type of stems demonstrated that the incidence of stress shielding was much higher (87.4–100%), and the grade of stress-shielding was more advanced [28–30]. In the previous study of load-stress analysis of TNS alloy stem using finite element methods showed that TNS alloy stem increased the load-stress in the cortical bone and might solve the load-stress imbalance between the cortical bone and prosthesis stems [21]. In the present study, the Kappa coefficient in the assessment of stress shielding was good. Previous study reported that the reproducibility of the evaluation of stress shielding was moderate among the cases with mild stress shielding compared [31]. There were no cases with advanced stress shielding in the present study, therefore it was reasonable the Kappa coefficient was resulted in not excellent, but good value.

The clinical scores of TNS alloy stem at 3 years in this study was significantly improved compared with the clinical scores at baseline. Previous clinical studies using the conventional titanium alloy (Ti-6Al-4V) stem demonstrated excellent results in the improvements of clinical scores [3–7]. We were not surprised to know that the mid-term results were comparable between the new TNS alloy stem and the conventionalTi-6Al-4V alloy stem in terms of clinical score improvement. However, in the long-term postoperative course, the TNS alloy stem may suppress the occurrence of stress-shielding and bone atrophy, which may contribute to a better clinical outcome in the long-term. Furthermore, considering these potential advantages, the TNS alloy stem may contribute to extend the durability of THA prosthesis and reduce the risk of revisional surgery.

The TNS alloy has a gradient functional property that allows us to change the modulus and strength by heat treatment, and this technology was applied for the stem used in this study. With the low thermal conductivity of TNS alloys, it is speculated that this functional gradient property could be applied to short designed stems used in anterior lateral supine hip approaches. In addition, the low Young’s modulus of TNS alloys may also be useful in other orthopaedic implants. Although locking plates with Ti-6Al-4V alloy for fracture treatment are widely used, the previous reports described that their excessive stiffness might inhibit an adequate micromotion for fracture healing and obstruct bone healing [32, 33]. In the previous studies using mice and rabbits tibial fracture models, the TNS alloy promoted bone healing and improved bone strength after healing compared to the Ti-6Al-4V alloy [34, 35]. Similarly, experiments in mice showed that TNS alloy intramedullary nails promoted bone healing better than stainless steel intramedullary nails [36]. In these studies, it was speculated that the TNS alloy adjusted the load-stress during bone healing and provided appropriate micromotion to the fracture site. The effect of TNS alloy on the adjustment of load-stress to promote bone healing seems to be consistent with the stress shielding prevention observed in this study. We believed that the TNS alloy is a promising material for orthopaedic implants.

To improve the biocompatibility of TNS alloy, the previous studies indicated the effect of anodic oxidation with acetic acid and sulfuric acid which demonstrated improved osseointegration with hydroxyapatite formation in experimental models [37–41]. In contrast, anodic oxidation of TNS alloy with sodium tartrate indicated photocatalytic activity with ultraviolet light irradiation [41]. Therefore, anodic oxide of TNS alloy prepared in sodium tartrate electrolyte may have the antibacterial effect using photocatalytic activity with ultraviolet light irradiation to prevent surgical site infection. Furthermore, the anodized TNS alloy prepared in sodium tartrate electrolyte had improved wear resistance while maintaining its elastic modulus [42, 43]. This seems to be another advantage of TNS alloy.

The present study had several limitations. First, there was no control in this study. The comparison between control cases and cases with TNS alloy femoral stems was not performed in this study because there were few cases operated using Ti-6Al-4V alloy femoral stems with similar design in the study period. Ideally, a cementless femoral stem made of Ti-6Al-4V alloy with the same shape should be used as a control. Second, the number of participants was small, and there was no process for minimizing selection bias in the selection of cases with TNS alloy femoral stem. Third, the follow-up period was short. Future studies are needed to clarify the usefulness of femoral stem of the TNS alloy in a larger cohort study with a long-term follow-up.

## Conclusion

The current study using a new TNS alloy femoral stem showed good clinical outcomes at 3-year follow-up. Radiologically, there was no loosening or subsidence of the stem. The mild stress shielding was observed in 65% of patients. No safety issues occurred during the study period. TNS alloys may be useful as a material for femoral stem.

## Data Availability

All data generated or analyzed during this study are included in this published article.

## Abbreviations

HV: Hardness of Vickers
JOA: Japanese Orthopaedic Association
JHEQ: Japanese orthopaedic association Hip disease Evaluation Questionnaire
THA: Total Hip Arthroplasty
TNS: Ti-Nb-Sn
RM: Robert Mathys
